# Development and experimental validation of analytical models for water and mud inrushes through a filled karst conduit

**DOI:** 10.1038/s41598-024-65930-4

**Published:** 2024-06-28

**Authors:** Xingjie Wu, Xuxu Yang, Hongwen Jing

**Affiliations:** 1 Shandong Key Laboratory of Civil Engineering Disaster Prevention and Mitigation, Shandong University of Science and Technology, Qingdao, 266590 China; 2 College of Civil and Architecture Engineering, Chuzhou University, Chuzhou, 239000 China; 3 State Key Laboratory for Geomechanics and Deep Underground Engineering, China University of Mining and Technology, Xuzhou, 221116 Jiangsu China

**Keywords:** Filled karst conduit, Water inrush, Mud inrush, Local instability model, Integral sliding instability model, Natural hazards, Civil engineering

## Abstract

Water or mud inrush has become a common geological disaster during tunnel construction in karst areas. To study forming process and mechanism of water and mud inrushes through a filled karst conduit, water inrush and mud inrush model tests were carried out with a self-developed 3D model test system. The results show that the forming processes of water inrush and mud inrush have different forming modes. For water inrush, the forming process follows: flowing instability of filling material particles—formation of water inrush channel—water inrush occurring; while for mud inrush, the forming process follows: stability—sliding instability of the whole filling material suddenly—mud inrush occurring. Accordingly, a local instability model of critical hydraulic pressure causing water inrush and an integral sliding instability model of critical hydraulic pressure causing mud inrush were established respectively. The two analytical models reveal the mechanism of water inrush and mud inrush experiments to an extent. The calculated critical hydraulic pressures for water inrush and mud inrush are in good agreement with the test results. The distinguishment of water inrush and mud inrush through a karst conduit was discussed based on the critical hydraulic pressure and the evolution law of seepage water pressure in tests, and a criterion was given. The research results might provide guidance for the forecast of water and mud inrush disasters during the construction of tunnels in karst area.

## Introduction

**Figure 1:**
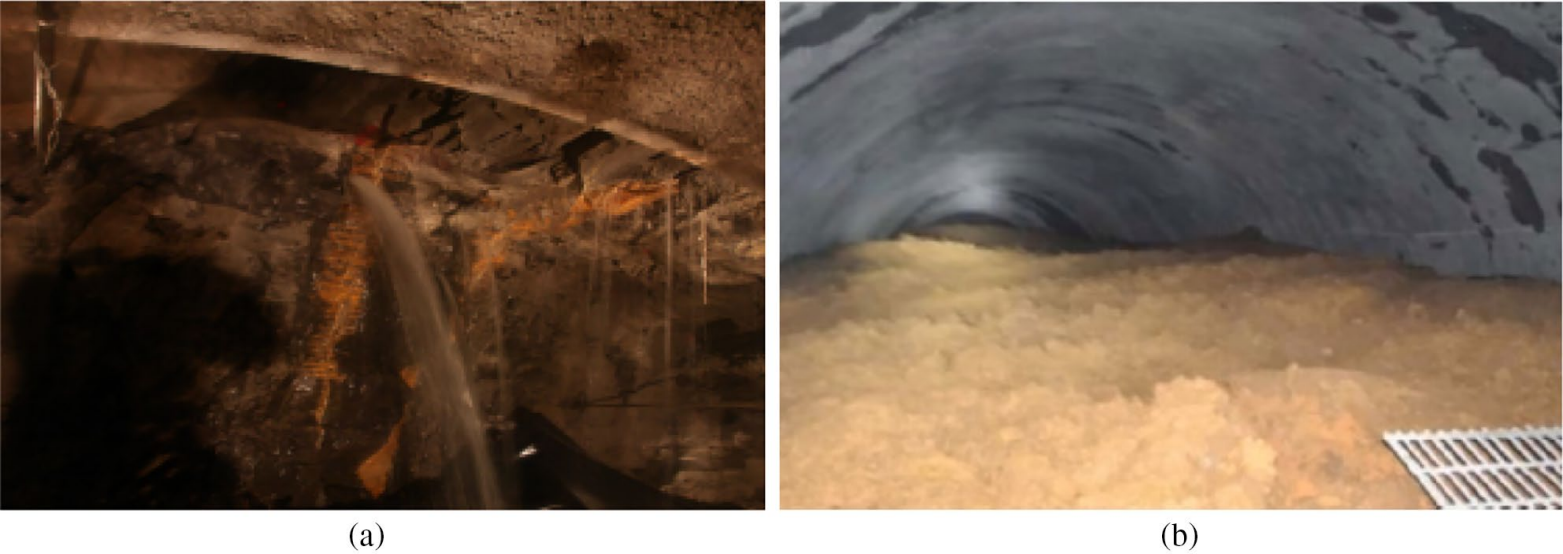
Water and mud inrushes during tunnel excavation. (**a**) Water inrush from drilling of boreholes in Jigongling tunnel (**b**) Mud inrush in Lingjiao tunnel.

With the development of express highways, high-speed railways, hydropower stations and other civil engineering projects, a large number of tunnels have been under construction in recent years^1,2^. Inevitably, the construction of tunnels is risky when encountering karst landforms, which account for one-third of China’s land area^3^.Water and mud inrushes have become common geological disasters that occur in karst regions^4^. Especially the karst conduit structure in these regions potentially has a strong ability to transport underground water and sediments^5^. For examples, several karst conduits were exposed during Jigongling tunnel construction, thereby leading to a large water inrush (Fig. 1a) with maximum water flux being 108 m^3^/h^6^. During the construction of Lingjiao tunnel, an extremely large mud inrush was formed because of blasting. Approximately 40,000 m^3^ deposits were subsequently accumulated at a distance of 190 m in the tunnel (Fig. 1b)^7^. Thus, water and mud inrush hazards during tunnel construction in karst regions have attracted great attention from researchers and engineers^8,9^.

Geomechanical model testing is one of the most important methods to simulate the process of water and mud inrushes for tunnels that are excavated in karst area. Shi et al.^10^ studied the influence of kaolin content and seepage loading rate on the seepage characteristics of filling medium with a large-scale triaxial stress-seepage test system. The seepage instability process of filling medium can be categorized into three stages, and the seepage failure process rate is proportional to the content of kaolin. Zhao et al.^11^ developed a simulated testing system for analyzing water–sand inrush through a vertical karst conduit. The test result shows that when the water pressure was close to the critical head pressure of the water–sand inrush, the water–sand inrush exhibited a pattern of instability—migration—deposition—stability. Li et al.^12^ studied the stability of the surrounding rock under the effect of karst cave with a true triaxial geomechanical model test based on the engineering background of the Xiema Tunnel. The results show that some internal connections exist among various information, and a theoretical model for prediction of water inrush in tunnels had been established. Wang et al.^13^ studied the hydraulic characteristics and disaster evolution of filling materials during water inrush under the influence of excavation. The permeability of karst conduit filling materials has an obvious impact on the development process of pore water pressure, as well as the zone and degree of excavation disturbance.

Moreover, theoretical analysis can further explore the formation mechanism of water inrush and mud inrush hazards. Zhu et al.^14^ presented a catastrophe theory-based risk evaluation model of water and mud inrush for tunnel excavation in karst area. The Qiyueshan tunnel of Yichang-Wanzhou railway was taken as an example, in which four target segments were evaluated using the risk evaluation model. Li et al.^15,16^ proposed a new slice-based method for calculating the minimum safe thickness for a filled-type karst cave. For intact and fractured resistant bodies, theoretical formulas for the minimum safe thickness were deduced from the tension strength and shear strength criteria of the rocks, respectively. Lin et al.^17,18^ interpreted the complex system of the tunnel, karst cave and filled media, and proposed an innovative method for the integral sliding stability analysis of the filling media on the basis of the simplified Bishop method. The influence of hydraulic effect, shear strength parameters, mud content in cave and boundary constraint on the global stability of fillings was researched. Chu^19^ established three types of filling karst conduit mechanical instability model considering the geological defects for karst water and mud inrush tectonic. And a water and mud inrush criterion was obtained by deducing the mechanical analysis.

Scholars have done a lot of research that benefits understanding the hazards of water and mud inrush in karst tunnels. However, for filled karst conduit geological structure, the process and difference of formation for water inrush and mud inrush are not clear yet, and the formation mechanism of the hazard is not fully understood. In this paper, in order to reveal the forming process and formation mechanism of water inrush and mud inrushes through a filled karst conduit, the process of water inrush and mud inrush hazards were reappeared respectively with a self-developed 3D model test system. According to the model test results, the forming modes of both hazards were analyzed, and a local instability model and an integral slipping model were further established. Based on the analysis of the two models, the different formation mechanisms and theoretical criteria of water inrush and mud inrush hazards were revealed. The outcomes may also help to better understand internal erosion and fines transportation flows through porous media in various industries, including but not limited to the prediction of sand production in oil and natural gas industry^20,21^, the suffusion process of granular soils in geological structures^22^.

## Simplified model and physical model tests

### Simplified model and design of test sample

**Figure 2:**
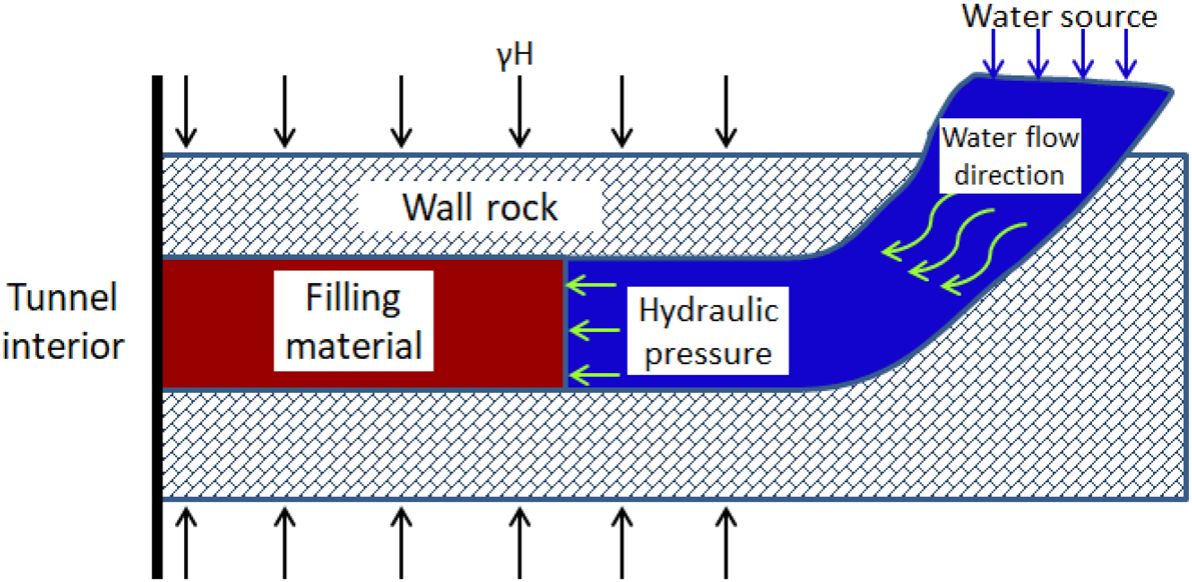
Simplified model of a filled karst conduit under hydraulic pressure.

Whether the water and mud inrush hazards occur mainly depends on the filling material and the hydraulic pressure. A simplified karst conduit model is provided to simulate a karst conduit connected to water source as shown in Fig. 2. The model could show the formation process of water and mud inrush hazards and the seepage water pressure in the filling material. The formulation of this model is based on the following assumptions: (1) The conduit is simplified into cuboid; (2) The stiffness of the filling material is much smaller than that of the conduit wall rock, so the deformation of the conduit wall rock is not considered.

**Figure 3:**
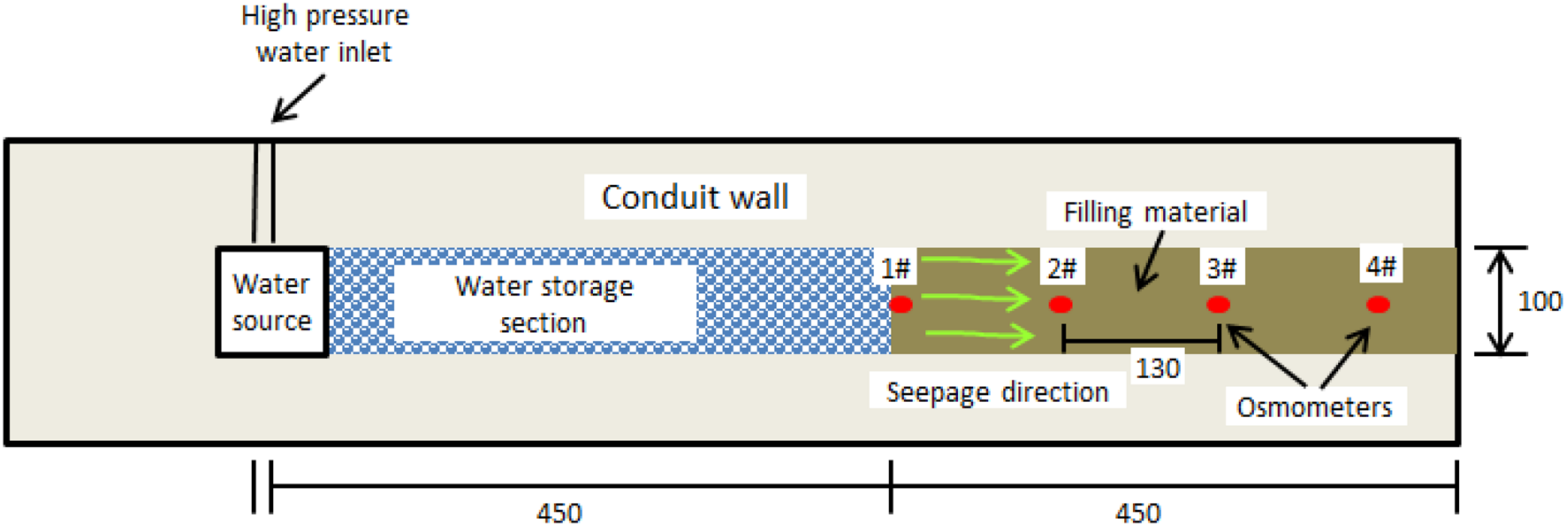
Design of test model (unit: mm).

Referring to the simplified model (Fig. 2), the test model (Fig. 3) is designed as a cuboid with a size of 30 cm × 30 cm × 180 cm. The cross-section shape of karst conduit is designed as a square with a side length of 100 mm. The model is divided into water source, water storage section and filling section from the middle to the end. And four osmometers are uniformly placed in the monitoring points.

### Test procedures

The conduit wall rock similar material is prepared with river sand, Vaseline, cement and water. And the composition ration of each composition material is 5:0.15:1:0.7. Conduit filling material is composed of clay and river sand, and two kinds of filling materials (Material A and Material B) are prepared. The properties of filling materials are listed in Table 1. The cohesion and internal friction angle of filling materials were measured by triaxial compression test.

**Table 1:** Permeability coefficient of mixing with different proportioning schemes.

Filling material	Mix. Ratio (clay: river sand)	Cohesion (kPa)	Internal friction angle (°)	Porosity	Permeability (× 10^−5^ cm/s)
Material A	1:0.8	45	31.8	0.15	1.06
Material B	1:0.6	58	28	0.12	0.53

**Figure 4:**
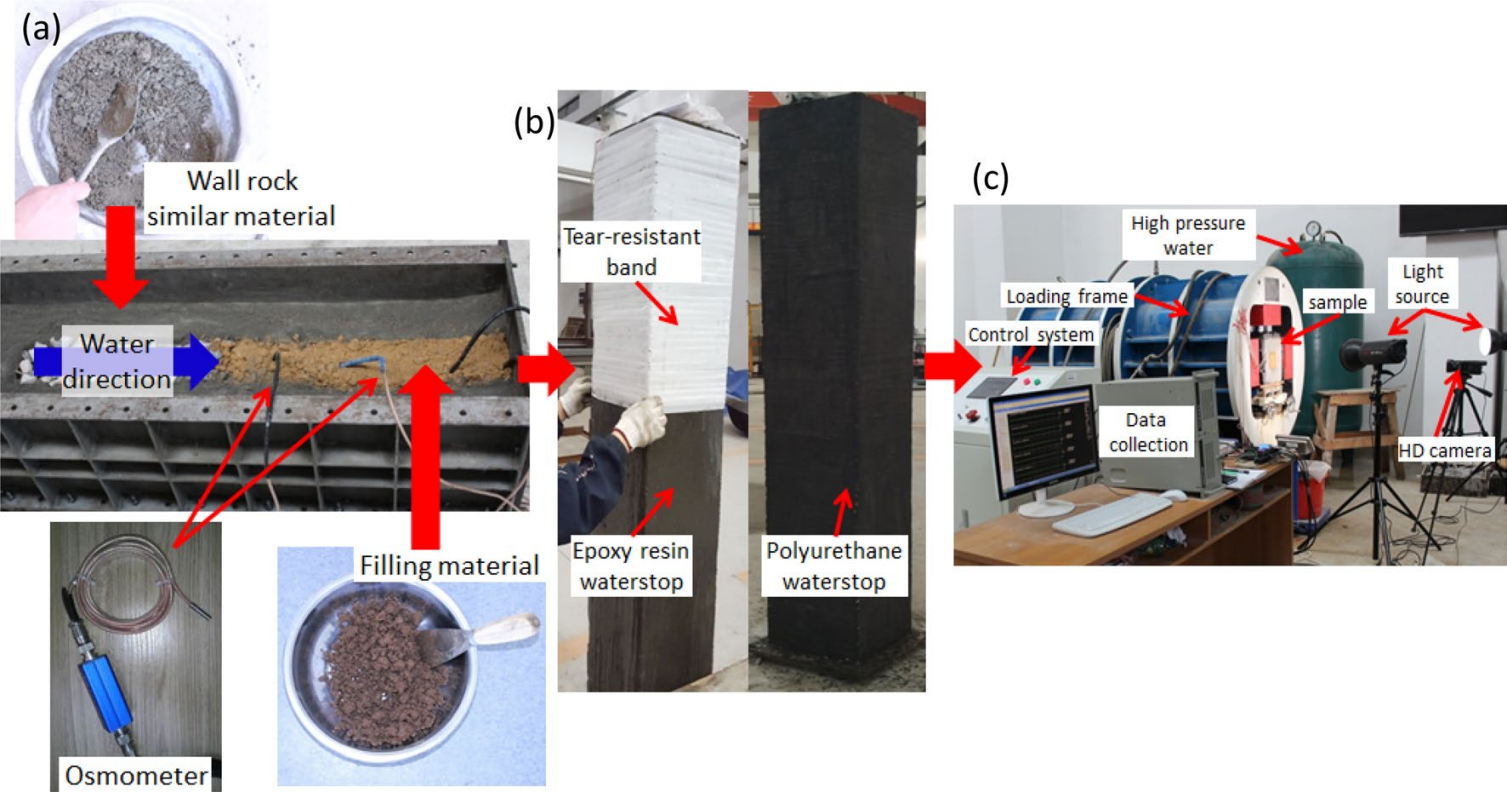
Test sample casting and test procedure: (**a**) Cast of sample; (**b**) Water-proof layers; (**c**) Test system.

According to the model layout scheme shown in Fig. 3, the model is laid and compacted evenly with conduit wall rock similar material and filling materials as shown in Fig. 4. Four osmometers are buried in the 1#-4# measuring points in the filling material (Fig. 4a). After 7 days’ curing, the boundary waterproofing system of the model including epoxy resin waterstop, tear-resistant band and polyurethane waterstop is set (Fig. 4b). A self-developed 3D model test system (Fig. 4c) was utilized to carry out the tests and the hydraulic pressure was loaded from 0.1 MPa with an interval of 0.1 MPa until water or mud inrush occurred. After each stage of hydraulic pressure loading, the seepage process in the filling material gradually stabilized, and then the next stage of hydraulic pressure loading was carried out.

### Test results and analysis

#### Water inrush hazard

**Figure 5:**
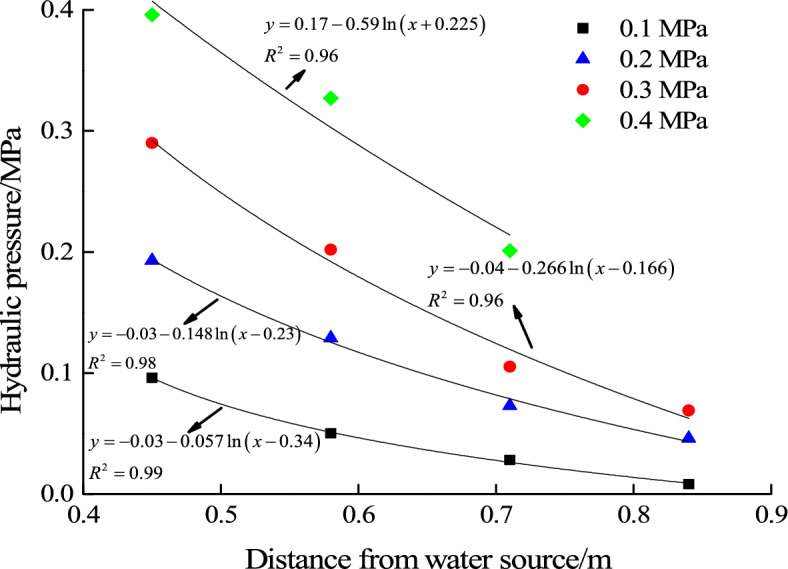
Seepage pressure evolution law of filling Material A (1:0.8).

For filling material A, the ratio of clay to river sand is 1:0.8 and water inrush hazard occurs in this test. During the hydraulic pressure loading process from 0.1 to 0.4 MPa, the seepage water pressure of each measuring point inside the filling material is shown in Fig. 5. The evolution law of water pressure along the seepage direction under each level of hydraulic pressure loading is also obtained by fitting.

**Figure 6:**
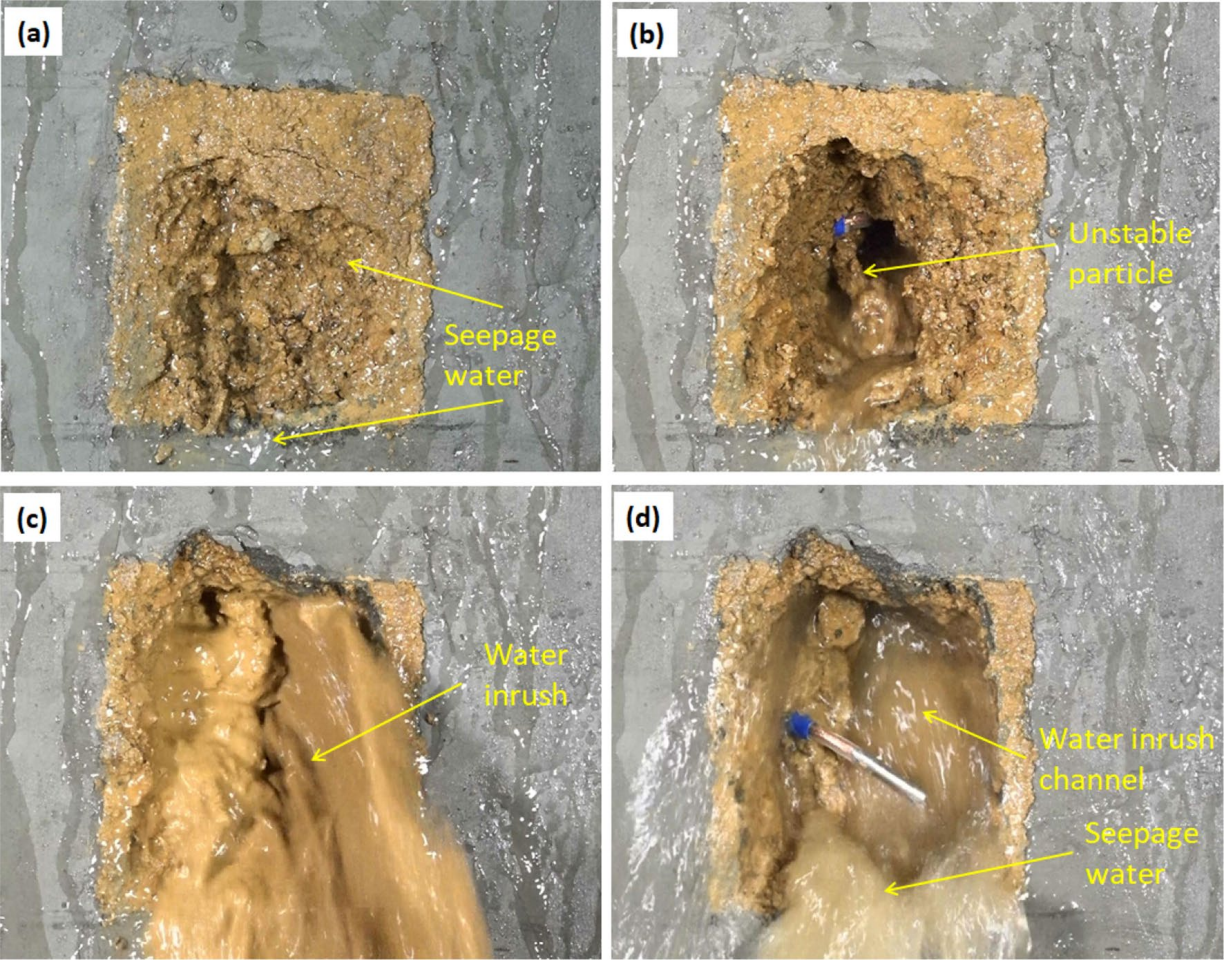
Formation process of water inrush. (**a**) Seepage stage, 0.1 MPa (**b**) Particles instability stage, 0.2 MPa (**c**) Channel formation stage, 0.3 MPa (**d**) Water inrush stage, 0.4 MPa.

The formation process of water inrush hazard is shown in Fig. 6. The water seeps to the free surface of filling material with the hydraulic pressure of 0.1 MPa (Fig. 6a). When the hydraulic pressure increases to 0.2 MPa, the filling material starts to produce instability in the form of particles and gradually extends inward from free surface (Fig. 6b). With the increase of hydraulic pressure to 0.3 MPa, water inrush channel inside the filling material is basically formed, and stable water inrush phenomenon is produced (Fig. 6c). As the hydraulic pressure continues to load, the water inrush channel further expands to periphery (Fig. 6d).

#### Mud inrush hazard

**Figure 7:**
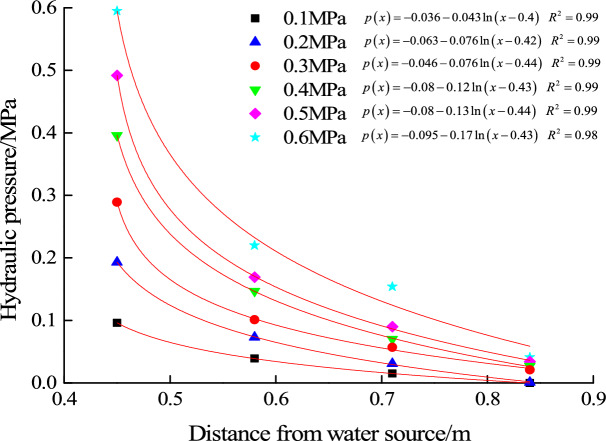
Seepage pressure evolution law of filling material B (1:0.6).

For filling material B, the ratio of clay to river sand is 1:0.6. When the hydraulic pressure increases to 0.6 MPa, mud inrush hazard occurs in this test. Water pressure of each measuring point infilling material under the action of each level of hydraulic pressure is shown in Fig. 7. And the evolution law of seepage pressure is also fitted.

**Figure 8:**
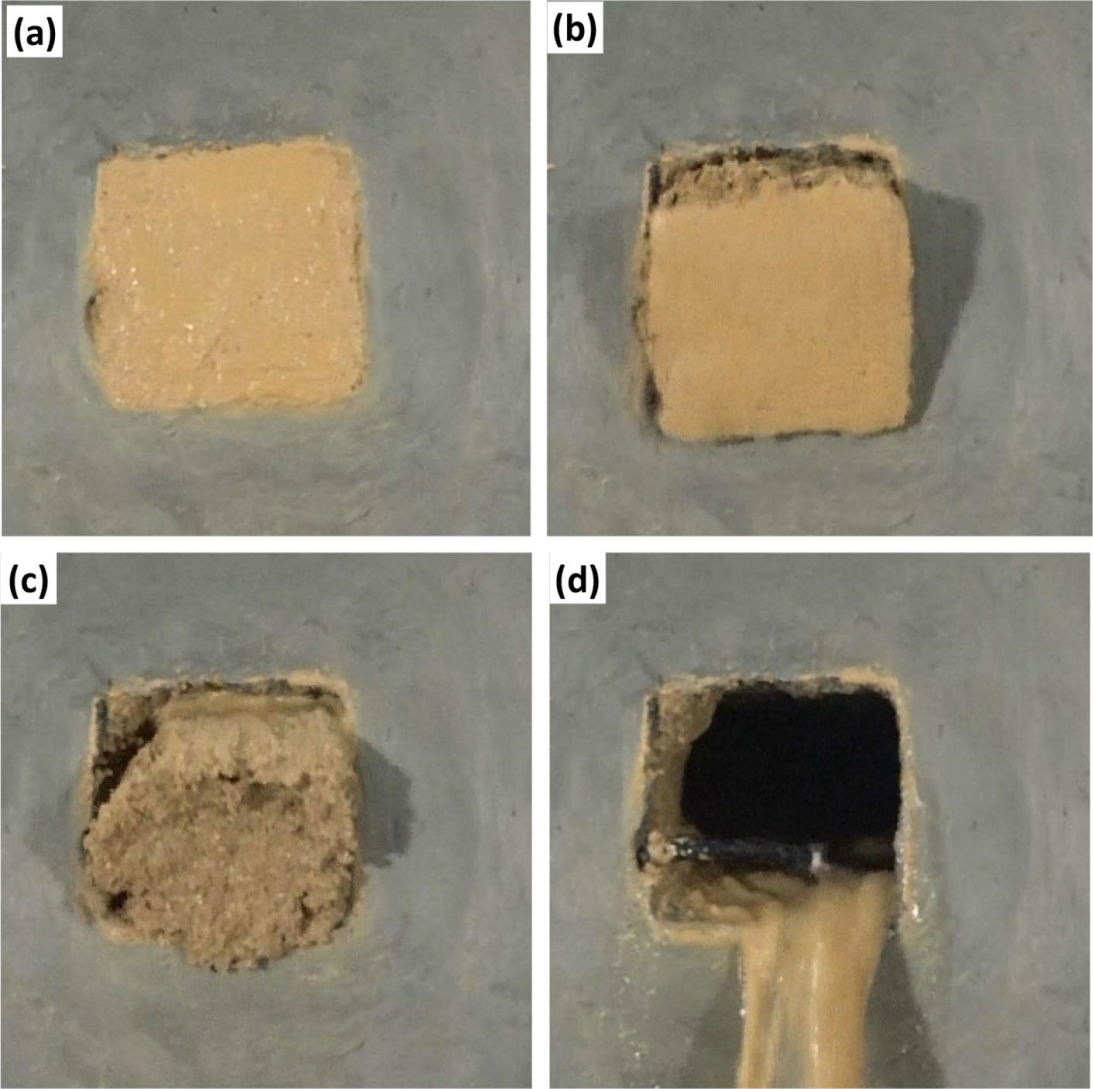
Formationprocess of mud inrush. (**a**) Stable stage, 0.1–0.5 MPa (**b**) Initial extrude stage, 0.6 MPa (**c**) Suddenly sliding stage, 0.6 MPa (**d**) End stage, 0.6 MPa.

The formation process of mud inrush hazard is shown in Fig. 8. In the process of hydraulic pressure increasing from 0.1 to 0.5 MPa, there is no other change for filling material except slightly wetting on the free surface (Fig. 8a).When the hydraulic pressure increased to 0.6 MPa, the filling material was found to extrude and move outward obviously (Fig. 8b). Then the whole filling material slides out along the conduit suddenly (Fig. 8c).And almost all the filling material in the conduit is extruded by the hydraulic water (Fig. 8d).

## Establishment and analysis of mechanical model

### Local instability model of filling material

**Figure 9:**
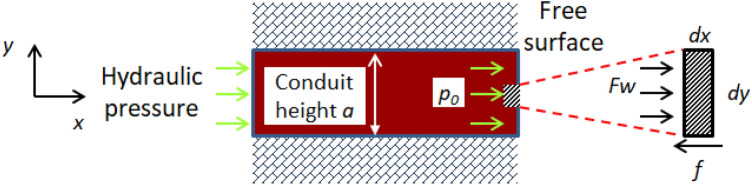
Scheme of local instability model.

In the water inrush test, the filling material particles gradually lost from the free surface to the inside and the water inrush channel gradually extends circumferentially. For water inrush formation process, taking the unit thickness perpendicular to the xy plane, a local instability model is established and the stress analysis of a small unit in the free surface of filling material is carried out as shown in Fig. 9. The model is based on following assumptions: (1) The filling material is elastic and isotropic material; (2) The weight of the filling material is not considered.

Assuming that the hydraulic gradient to the small unit is $i$, the water pressure on the filling material at the free surface is

1 $$F_{w} = \int\limits_{{ - \frac{a}{2}}}^{\frac{a}{2}} {\int\limits_{x}^{x + dx} {n\,\gamma \,_{w} i\,} } dx\,dy = n\,\gamma_{w} \,i\,a\,dx$$

where $n$ is the porosity of the filling material.

Marking the cohesion of the filling material is $c$, and the dragging force generated by the surrounding filling material is

2 $$f = 2cdx$$

When the filling material at free surface is in the limit equilibrium state, there is an equilibrium equation $F_{{\text{w}}} = f$. And the equation is simplified to get

3 $$i = \frac{2c}{{n\gamma_{w} a}}$$

The water pressure acting on the small unit is $p_{0}$ and the average particle size of the filling material is $d$, there is a relationship $i = \frac{{p_{0} }}{{\gamma_{w} d}}$ and put it into Eq. (3). The critical hydraulic pressure at free surface when the filling particles are unstable is

4 $$p_{0} = \frac{2\,cd}{{na}}$$

### Integral sliding instability model

**Figure 10:**
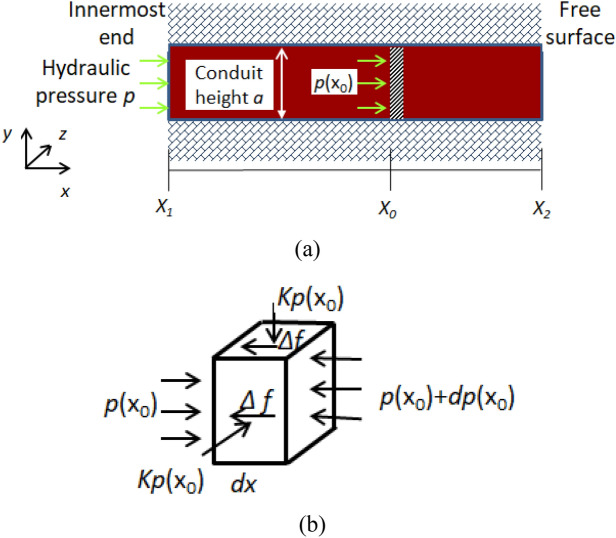
Model of integral sliding instability. (**a**) Sketch of model (**b**) Force analysis unit.

For the mud inrush formation process, an integral sliding instability model (Fig. 10a) is established based on the following assumptions: (1) The cohesion between the filling material and the conduit wall is not considered, while the friction between them is considered; (2) The filling material is elastic and isotropic material; (3) The weight of the filling material is not considered, and the filling material only deforms in the x direction during mud inrush process.

A small unit at position $x_{0}$ is taken as the research object. The stress state of the small unit is shown in Fig. 10b. Due to the overall slip instability of the filling material, the sizes of three dimensions of the conduit are considered. The water pressure of the small unit in the $x$ direction is $p(x_{0} )$. The pressure generated by hydraulic is

5 $$F = p\left( {x_{0} } \right)a^{2}$$

The pressure between the filling material and the surrounding conduit wall is $Kp(x_{0} )$, where $K$ is the lateral pressure coefficient. According to generalized Hooke’s law, the strain in the y and z directions can be expressed as

6 $$\left\{ {\begin{array}{*{20}c} {\varepsilon_{y} = \frac{1}{E}\left[ {\sigma_{y} - \mu \left( {\sigma_{z} + \sigma_{x} } \right)} \right]} \\ {\varepsilon_{z} = \frac{1}{E}\left[ {\sigma_{z} - \mu \left( {\sigma_{x} + \sigma_{y} } \right)} \right]} \\ \end{array} } \right.$$

Let $\varepsilon_{y} = \varepsilon_{z} = 0$, $\sigma_{y} = \sigma_{z} = \sigma^{\prime }$, we can get $\sigma^{\prime } - \mu \left( {\sigma^{\prime } + \sigma_{x} } \right) = 0$, and simplifies to be $\sigma^{\prime } = \frac{\mu }{1 - \mu }\sigma_{x}$. That is, the expression of the lateral pressure coefficient can be obtained as

7 $$K = \frac{\mu }{1 - \mu }$$

The sliding friction force generated between the small unit and conduit wall rock is

8 $$df\left( x \right) = 4aK\,\tan \varphi p(x_{0} )\,dx$$

Assuming that the sliding instability of the filling material begins at position $x_{0}$, the sliding friction force on the filling material from $x_{0}$ to $x_{2}$ is

9 $$f = \int_{{x_{0} }}^{{x_{2} }} {df\left( x \right)} = 4aK\tan \varphi \int_{{x_{0} }}^{{x_{2} }} {p(x)\,dx}$$

The equation can be established according to the force balance in the sliding instability section of the filling material

10 $$F = f$$

Substitute Eqs. (5) and (9) into Eq. (10),we can get the Eq. (11) after simplification.

11 $$p\left( {x_{0} } \right)a^{2} = 4aK\,\tan \varphi \int_{{x_{0} }}^{{x_{2} }} {p(x)\,dx}$$

In particular, when the full length of the filling material produces sliding instability, the hydraulic is $p(x_{0} ) = p$, and the critical hydraulic pressure for mud inrush is

12 $$p = \frac{4}{a}K\tan \varphi \int_{{x_{1} }}^{{x_{2} }} {p(x)dx}$$

## Verification

### Water inrush and local instability model

For Eq. (4), the conduit height $a$ in the test is 10^−1^ m. According to the water inrush test process, the unstable filling particles are centimeter-level when the water inrush channel formed, which is uniformly $d = 10^{ - 2} {\text{m}}$.

**Figure 11:**
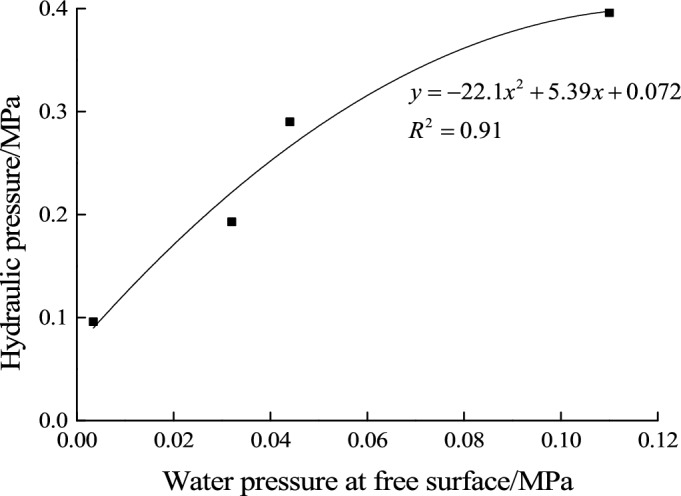
Fitting equation between water pressure at free surface and hydraulic pressure.

According to the equation of seepage water pressure inside the filling material fitted in the test, water pressure at free surface can be calculated as shown in Table 2. The relationship between hydraulic pressure and water pressure at free surface can be fitted and the result is shown in Fig. 11.

**Table 2:** Water pressure at free surface under different hydraulic pressure.

Filling material type	Material A			
Hydraulic pressure (MPa)	0.1	0.2	0.3	0.4
Water pressure at free surface (MPa)	0.003	0.032	0.044	0.11

In the water inrush hazard model test, the test result shows that when the hydraulic pressure reaches to 0.3 MPa, the water inrush channel formed already. So the experimental critical hydraulic pressure is 0.3 MPa in water inrush model test.

The theoretical water pressure at free surface when water inrush occurs can be calculated with Eq. (4). Further, the corresponding theoretical critical hydraulic pressure can be obtained by the equation in Fig. 11.The calculation results of water inrush test are shown in Table 3. By comparing the theoretical and experimental results of the critical hydraulic pressure for water inrush, it is found that the deviation between them is 5.3%, which proves that the local instability model is reasonable.

**Table 3:** Comparison table of experimental and theoretical hydraulic pressure for water inrush.

Filling material type	Experimental critical hydraulic pressure (MPa)	Calculated free surface water pressure (MPa)	Theoretical critical hydraulic pressure (MPa)	Deviation
A (1:0.8)	0.3	0.06	0.316	5.3%

### Mud inrush hazard and integral sliding instability model

The theoretical critical hydraulic pressure when mud inrush hazard occurs can be calculated according to Eq. (12). The calculation parameters are shown in Table 4.

**Table 4:** Calculation parameters of mud inrush hazard.

$x_{1}$(m)	$x_{2}$(m)	Conduit size $a$ (m)	Poisson’s ratio	Lateral pressure coefficient	Friction angle (°)	Friction coefficient	Cohesion $c$ (kPa)
0.45	0.9	0.1	0.25	1/3	28	0.53	58

One of the most important part in Eq. (12) is the seepage water pressure evolution formula $p\left( x \right)$ in filling material. Perazzelli et al.^23^ put forward the distribution formula of height of water head in tunnel surrounding rock, which is

13 $$h\left( {x,y,z} \right) = h_{F} + \left( {1 - e^{{ - b\frac{x}{H}}} } \right)\Delta h$$

where $H$ is the height of the tunnel, and $h_{F}$ is the piezometric head applied to the tunnel face from inside the tunnel, and $\Delta h$ is the difference between the elevation of the water table $h_{0}$ and the hydraulic head $h_{F}$, and $b$ is the coefficient which can be obtained by fitting.

Here we only consider the law of seepage water pressure in the $x$ direction. In addition, the end face of the filling material in the test is free surface, then there is a relation $h_{F} = 0$. At the same time, considering the difference between the coordinate system established in this paper and reference^23^, the corresponding adjustment should be made to Eq. (13). The water head height should be replaced by water pressure, and then the evolution equation of the seepage water pressure inside the filling material along the seepage path in the mud inrush test can be obtained as

14 $$p\left( x \right) = e^{{ - b\frac{{x - x_{1} }}{a}}} p$$

where, $p$ is the hydraulic pressure loaded. The seepage water pressure at 2#, 3# and 4# position can be fitted to obtain the coefficient $b$, and the results are shown in Table 5.

**Table 5:** Calculation table of coefficient $b$.

$x$ position (m)	Seepage water pressure (MPa)	Coefficient $b$	Average of coefficient $b$
0.58	0.22	0.765	0.66
0.71	0.154	0.523	
0.84	0.041	0.688	

By substituting Eq. (14) into Eq. (12) and using the parameters in Table 4, the theoretical critical hydraulic pressure for mud inrush can be calculated as shown in Table 6. By comparing the experimental and the theoretical results, it is found that the deviation is 1.7%, which shows the reasonability of integral sliding instability model.

**Table 6:** Comparison table of experimental and theoretical hydraulic pressure for mud inrush.

Experimental critical hydraulic pressure (MPa)	$p\left( x \right)$	Theoretical critical hydraulic pressure (MPa)	Deviation
0.6	$p\left( x \right) = e^{{ - 6.6\left( {x - 0.45} \right)}} p$	0.61	1.7%

## Discussion

Many scholars have studied the identification of water inrush and mud inrush. However, it is mainly classified from the perspective of geological features and material types after hazards occur^24^. This part attempts to predict the types of water inrush and mud inrush hazards through filled karst conduit from the perspective of mechanics before the disaster occurs, so as to contribute to disaster prediction and tunnel construction safety protection for karst tunnels.

**Figure 12:**
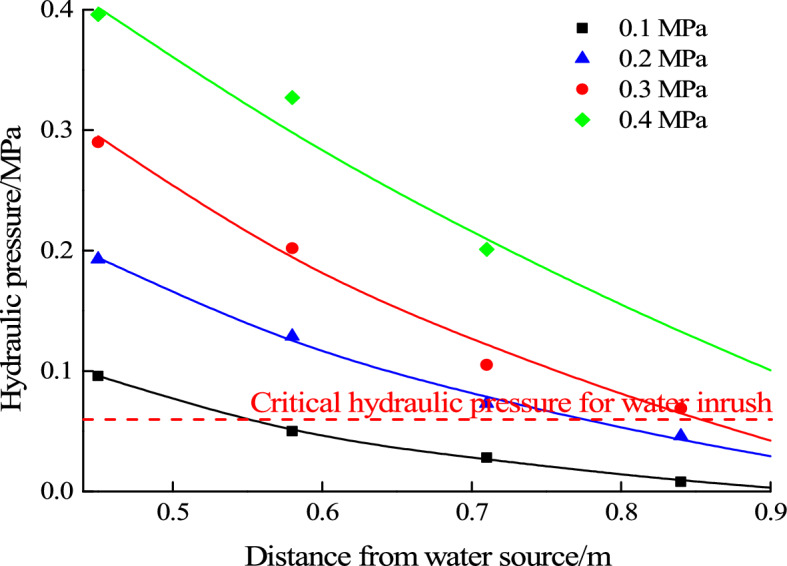
Relationship between seepage water pressure and critical pressure for water inrush.

Figure 12 shows the relationship between the law of seepage water pressure evolution in water inrush test and the critical water pressure at free surface for water inrush. It can be seen that when the hydraulic pressure is 0.1 MPa and 0.2 MPa, water pressure at the free surface is much lower than the critical water pressure. When the hydraulic pressure increases to 0.3 MPa, it is close to the critical water pressure. When the hydraulic pressure is 0.4 MPa, it is much higher than the critical water pressure. Considering the forming process of water inrush, it can be seen that when the water pressure at free surface is close to the critical value, the water inrush channel is gradually formed. When the water pressure at free surface is higher than the critical value, the water inrush channel continues to expand. From the previous analysis, it can be seen that when the water pressure at free surface reaches the critical value, the filling material particles begin to become unstable and water inrush channel gradually forms. And eventually water inrush hazard occurs.

**Figure 13:**
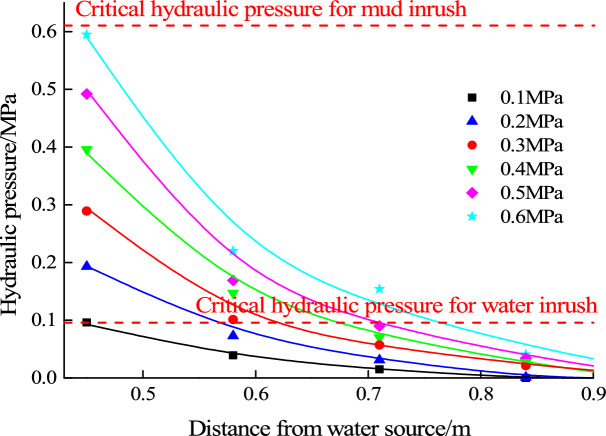
Relationship between seepage water pressure and critical pressure for mud inrush.

The relationship between seepage water pressure and critical hydraulic pressure for mud inrush is shown in Fig. 13. With the loading of hydraulic pressure, the water pressure at the innermost end of the filling material is the largest. And when the water source pressure is loaded to 0.6 MPa, the water pressure at the innermost end of the filling material is close to the critical hydraulic pressure for mud inrush. In mud inrush test, it is observed that the whole filling material starts to slid out of the karst conduit. At the same time, it is found that the seepage water pressure at free surface is much lower than the critical pressure for water inrush, and the instability of the filling particles is not found during the mud inrush test. It can be concluded that the water pressure in the innermost end of the filling material gradually increases to the critical hydraulic pressure for mud inrush, which results in integral sliding instability, and eventually results in mud inrush hazard.

**Figure 14:**
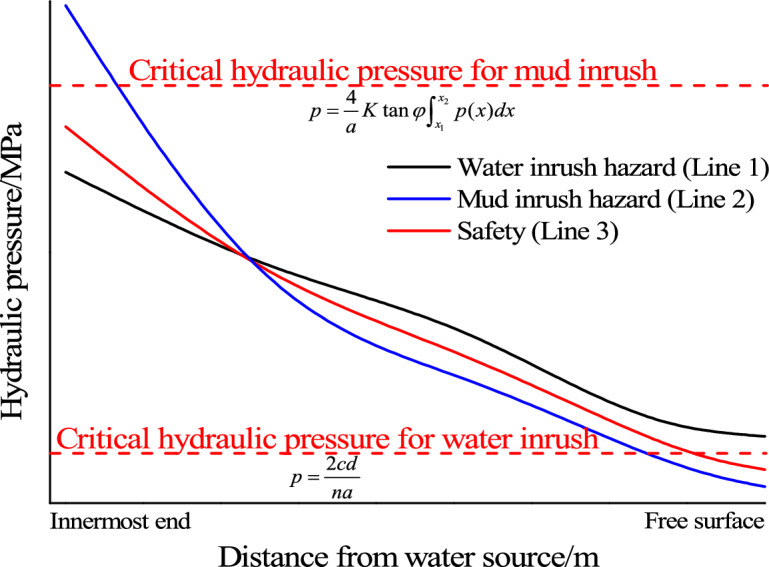
Prediction diagram of hazards type.

Given the diagram to predict the types of water inrush and mud inrush hazards as shown in Fig. 14, basing on the analysis of Figs. 12 and 13. The three curves in the figure are conceptualized evolution laws of seepage water pressure in the filling material. According to the relationship between seepage water pressure and critical hydraulic pressure for water inrush and for mud inrush, hazard types can be predicted by the following criteria: (1) If seepage water pressure at free surface is higher than the critical hydraulic pressure for water inrush (for example Line 1), filling material particles will gradually be instability from the free surface and gradually form a water inrush channel. And finally a water inrush hazard will occur. (2) When the maximum seepage water pressure in filling material exceeds the critical hydraulic pressure for mud inrush (for example Line 2), the filling material will slip to the free surface, and eventually form a mud inrush hazard. (3) When the seepage water pressure at free surface is lower than the critical hydraulic pressure for water inrush, and the inner water pressure is less than the critical hydraulic pressure for mud inrush, neither the filling material at free surface will form a water inrush channel, nor the filling material will slid out (for example Line 3).

## Conclusions

To study the forming process and mechanism of water and mud inrush hazards through a filled karst conduit, laboratory tests were carried out with a self-developed 3D model test system. The process of water inrush and mud inrushes was reappeared in the model tests respectively. A local instability model and an integral sliding instability model are established for different forming mode. The results in this study could be summarized as follows:

1. According to the forming process of hazards in model tests, it is found that water inrush and mud inrush show different forming modes. For water inrush, the filling material in the form of small particles starts to become unstable from the free surface. Then a water inrush channel is formed gradually and water inrush hazard occurs finally. For mud inrush, the filling material as a whole becomes unstable and slides out from the karst conduit suddenly, resulting in mud inrush hazard.
2. According to different forming modes, a local instability model is established for water inrush hazard, and an integral sliding instability model for mud inrush hazard. The mathematical formula of critical hydraulic pressure causing water inrush and mud inrush are obtained respectively. And for integral sliding instability model, the influence of conduit size, lateral pressure coefficient and friction angle on the critical hydraulic pressure are analyzed. The theoretical results are compared with the test results and the deviation of both for local instability model is 5.3%, and that for integral sliding instability model is 1.7%.
3. Based on the theoretical critical hydraulic pressure and the evolution law of seepage water pressure in tests, the theoretical basis for the prediction of hazard typesis established, and the criterion for the prediction is given.

## Data Availability

The data presented in this study are available on reasonable request from the corresponding author.

## References

[1] Liu J, Zhao Y, Tan T, Zhang L, Zhu S, Xu F (2022). Evolution and modeling of mine water inflow and hazard characteristics in southern coalfields of China: A case of Meitanba mine. Int. J. Min. Sci. Technol..

[2] Chen L, Wang Z, Zhang W, Wang Y (2023). Characteristics, numerical analysis and countermeasures of mud inrush geohazards of Mountain tunnel in karst region. Geomat. Nat. Haz. Risk..

[3] Lai J, Zhou H, Wang K, Qiu J, Wang L, Wang J, Feng Z (2020). Shield-driven induced ground surface and Ming Dynasty city wall settlement of Xi’an metro. Tunn. Undergr. Sp. Tech..

[4] Wang X, Li S, Xu Z, Hu J, Pan D, Xue Y (2019). Risk assessment of water inrush in karst tunnels excavation based on normal cloud model. B. Eng. Geol. Environ..

[5] Xue Y, Kong F, Li S, Qiu D, Su M, Li Z, Zhou B (2021). Water and mud inrush hazard in underground engineering: Genesis, evolution and prevention. Tunn. Undergr. Sp. Tech..

[6] Li S, Zhou Z, Li L, Xu Z, Zhang Q, Shi S (2013). Risk assessment of water inrush in karst tunnels based on attribute synthetic evaluation system. Tunn. Undergr. Sp. Tech..

[7] Li X, Zhang P, He Z, Huang Z, Cheng M, Guo L (2017). Identification of geological structure which induced heavy water and mud inrush in tunnel excavation: A case study on Lingjiao tunnel. Tunn. Undergr. Sp. Tech..

[8] Liu N, Pei J, Cao C, Liu X, Huang Y, Mei G (2022). Geological investigation and treatment measures against water inrush hazard in karst tunnels: A case study in Guiyang, southwest China. Tunn. Undergr. Sp. Tech..

[9] Kong F, Xue Y, Gong H, Jiang X, Song Q, Fu Y, Fu K (2023). The formation mechanism of dynamic water and mud inrush geohazard triggered by deep-buried tunnel crossing active fault: Insights from the geomechanical model test. Tunn. Undergr. Sp. Tech..

[10] Shi S, Zhu X, Cao Z, Bu L, Wen Z, Zhou Z, Guo W, Zhao R (2022). Experimental study of seepage characteristics of filling structures in deep roadway. Geofluids..

[11] Zhao Z, Wang H, Han L, Zhao Z (2023). Study of water-sand inrush through a vertical karst conduit uncovered through tunnel excavation. Water..

[12] Li S, Gao C, Zhou Z, Li L, Wang M, Yuan Y, Wang J (2019). Analysis on the precursor information of water inrush in karst tunnels: A true triaxial model test study. Rock Mech. Rock Eng..

[13] Wang Y, Zheng S, Zhong Z, Li Y, Li Z (2023). Experimental investigation on the hydraulic characteristics of water inrush in deep buried filled karst conduit considering the permeability. Transp. Geotech..

[14] Zhu J, Li T (2020). Catastrophe theory-based risk evaluation model for water and mud inrush and its application in karst tunnels. J. Cent. South Univ..

[15] Li S, Wang J, Li L, Shi S, Zhou Z (2019). The theoretical and numerical analysis of water inrush through filling structures. MathComput. Simulat..

[16] Xu Z, Huang X, Li S, Lin P, Shi X, Wu J (2020). A new slice-based method for calculating the minimum safe thickness for a filled-type karst cave. B. Eng. Geol. Environ..

[17] Lin, P., Li, S., Xu, Z., Li, L., Huang, X. & He, S. Analysis of stability of mud inrush induced by fillings sliding failure in karst cave based on the simplified Bishop method and its application. Geo-China: Emerging technologies in tunnel engineering, modeling, design, construction, repair, and rehabilitation. *Reston, Va. Am. Soc. Civ. Eng*. 73–80 (2016).

[18] Li S, Lin P, Xu Z, Li L, He S, Zhao S, Huang X (2017). Innovative method for the integral sliding stability analysis of filling media in karst caves and its applications in engineering. Int. J. Geomech..

[19] Chu V (2016). Mechanism on water inrush disaster of filling karst piping and numerical analysis of evolutionary process in highway tunnel. J. Cent. South Univ..

[20] Song Y, Zhang D, Ranjith PG, Zhou Z, Wu B, Kong L, Chen L, Huang J (2024). A comprehensive study of fines migration in internally unstable natural gas hydrate reservoirs. Powder Technol..

[21] Song Y, Ranjith PG, Wu B (2020). Development and experimental validation of a computational fluid dynamics-discrete element method sand production model. J. Nat. Gas Sci. Eng..

[22] Zhou Z, Ranjith PG, Li S (2018). An experimental testing apparatus for study of suffusion of granular soils in geological structures. Tunn. Undergr. Sp. Tech..

[23] Perazzelli P, Leone T, Anagnostou G (2014). Tunnel face stability under seepage flow conditions. Tunn. Undergr. Sp. Tech..

[24] Xue Y, Kong F, Qiu D, Su M, Zhao Y, Zhang K (2021). The classifications of water and mud/rock inrush hazard: A review and update. B. Eng. Geol. Environ..

